# Characterization of the pathogenicity of a *Bacillus cereus* isolate from the Mariana Trench

**DOI:** 10.1080/21505594.2022.2088641

**Published:** 2022-06-22

**Authors:** Yujian Wang, Jian Zhang, Zihao Yuan, Li Sun

**Affiliations:** a CAS and Shandong Province Key Laboratory of Experimental Marine Biology, Institute of Oceanology, Center for Ocean Mega-Science, Chinese Academy of Sciences, Qingdao, China; b Laboratory for Marine Biology and Biotechnology, Pilot National Laboratory for Marine Science and Technology (Qingdao), Qingdao, China; c College of Earth and Planetary Sciences, University of Chinese Academy of Sciences, Beijing, China; d School of Ocean, Yan tai University, Yantai, China

**Keywords:** *Bacillus cereus*, deep sea, pathogenicity, cytotoxicity, spore

## Abstract

*Bacillus cereus* is an important opportunistic pathogen widely distributed in the environment. In this study, we reported the isolation and characterization of a *B. cereus* isolate, MB1, from the Challenger Deep of the Mariana Trench. MB1 is aerobic, motile, and able to form endospores. It possesses 5966 genes distributed on a circular chromosome and two plasmids. The MB1 genome contains 14 sets of 23S, 5S, and 16S ribosomal RNA operons, 106 tRNA genes, 4 sRNA genes, 12 genomic islands, 13 prophages, and 302 putative virulence genes, including enterotoxins and cytolysins. Infection studies showed that MB1 was able to cause acute and lethal infection in fish and mice, and was highly toxic to mammalian cells. MB1 induced, in a dose-dependent manner, pyroptotic cell death, characterized by activation of caspase-1, cleavage of gasdermin D, and release of IL-1β and IL-18. MB1 spores exhibited swimming and haemolytic capacity, but were severely attenuated in pathogenicity, which, however, was regained to the full extent when the spores germinated under suitable conditions. Taken together, these results provide new insights into the biological and pathogenic mechanism of deep sea *B. cereus*.

## Introduction

*Bacillus cereus* is a Gram-positive bacterium and an opportunistic pathogen with clinical importance. It belongs to the *B. cereus* group, which contains at least 8 closely related species, including *Bacillus anthracis*, *Bacillus thuringiensis*, and *Bacillus toyonensis* [1]. These bacteria are exceedingly conserved in genetics and have highly similar 16S rRNA gene sequences, yet they can be defined as unique species based on their phenotypes and clinical or economic significance [2]. Some of these bacteria have been exploited for commercial purpose. For example, *B. thuringiensis* is used as an effective insecticidal agent, on the account that it harbours a plasmid that encodes a variety of insecticidal toxins [3,4]. Some of the *B. cereus* group species are recognized as important pathogens, e.g. *B. anthracis* can cause fatal human diseases. Some other *B. cereus* group members possess probiotic properties, e.g. *B. toyonensis* and *B. subtilis* can regulate the intestinal flora and therefore are beneficial to the host [5,6].

*B. cereus* was originally considered a harmless environmental pollutant, later it was found to be related to intestinal diseases, and now it is confirmed to be an important pathogen inducing serious and potentially fatal non-intestinal diseases [7,8]. As the causative agent of many foodborne diseases, B. cereus, ingested as viable cells or spores, produces and secretes enterotoxins that induce a series of food poisoning, primarily manifested as diarrhoea and emesis [2,9]. Two enterotoxin complexes, haemolysin BL (HBL) and nonhemolytic enterotoxin (NHE), are closely related to the diarrhoea-type food poisoning [10], while the vomiting-type is mainly caused by a dodecadepsipeptide, cereulide [11]. In addition to intestinal infections, B. cereus also causes severe non-gastrointestinal infections, including central nervous system infection, endocarditis, respiratory and urinary tract infections, endophthalmitis, and septicaemia, in both immunocompromised and immunocompetent individuals [12]. Recent studies showed that B. cereus infection of human monocytes and mouse macrophages rapidly activated the NLRP3 inflammasome, which then triggered gasdermin D (GSDMD)-mediated pyroptosis and secretion of the inflammatory cytokines IL-1β and IL-18 [13,14]. It is worth noting that this ability to induce inflammation through inflammasomes led to lethality in mice [15].

*B. cereus* is widely distributed in nature, including certain extreme environments, such as the deep sea. The Challenger Deep in the Mariana Trench is the deepest ocean on earth and shares comparable physical or chemical conditions with the abyssal layers [16]. It collects abundant suspended organic matters due to the funnelling effect, and forms a distinct microbial ecosystem driven by endogenous recycling of organic matters [17,18]. Several studies showed that Bacillus sp. is present in deep sea, and some Bacillus members occupy the dominant positions in cultivable deep-sea bacterial communities [19–21]. However, the toxic and pathogenic potentials of B. cereus from the Mariana Trench remain to be investigated.

In this study, we reported the isolation of a *B. cereus* strain, MB1, from the Challenger Deep of the Mariana Trench, and examination of its biological characteristics, genome feature, and pathogenicity. We found that MB1 possesses a large number of virulence associated genes including enterotoxins. Consistently, MB1 was highly cytotoxic and able to disseminate in fish and mice tissues with lethal consequence. Our results provide new insights into the pathogenicity of deep sea *B. cereus*.

## Materials and methods

### Isolation of MB1

Strain MB1 was isolated from a deep-sea water sample collected at −8,003 m in the Mariana Trench (Figure S1). The water sample was obtained using a gravity sampler on the lander equipped on the research vessel *Zhangqian*. For bacterial isolation, 100 μl of the seawater was spread on a marine 2216E agar (2216EA; Haibo, Qingdao, China) plate in an ultra-clean workbench and incubated at 28 °C under aerobic condition for 5 days. After three consecutive re-cultivations of selection and purification, a *Bacillus* isolate was obtained and named MB1. The strain was stored at −80 °C in marine 2216E broth medium supplemented with 30% (v/v) glycerol.

### Morphological and growth features of MB1

To observe the morphology of MB1 on the solid medium, MB1 was streaked in 2216EA plate and incubated at 28 °C overnight. The scanning electron microscope (SEM) and transmission electron microscope (TEM) observations were performed as previously reported [22]. Sporulation was induced by continuous culturing of MB1 in 2216E medium supplemented with 5 mg/L MnSO_4_ at 28 °C for more than 72 h. The spores were stained using a spore staining kit (Solarbio, Beijing, China), followed by observation with a microscope (Ti-S/L100, Nikon, Tokyo, Japan). MB1 growth at different temperatures, pH, and NaCl was examined as reported previously [23]. For the haemolysis assay, MB1 was cultured in marine 2216E medium to an OD_600_ of 0.8 and centrifuged at 8000 g. The bacterial cells and supernatant were collected after centrifugation. The cells were resuspended in PBS to 10^8^ CFU/mL. Ten microlitres of cell suspension, supernatant, and 2% TritonX-100 were each dropped onto filter discs in a 2216EA plate containing 5% sheep blood. The plate was incubated at 28 °C for 24 h and observed for haemolysis. For swimming analysis, 10 μl of the above MB1 suspension was dropped onto a 2216EA plate containing 0.3% (w/v) agar. The plate was incubated at 28 °C for 24 h and observed for bacterial motility.

### Animals and cell lines

Clinically healthy turbots (20 ± 1.87 g) were purchased from a local fish farm and maintained at 20 °C in aerated seawater. The fish were acclimatized in the laboratory for 2 weeks before experimental manipulation. BALB/c mice (female, 8 weeks, 18 ± 0.55 g) were purchased from Qingdao Daren Fortune Animal Technology Co., Ltd (Qingdao, China). For tissue collection, fish were euthanized with tricaine methanesulfonate (Sigma, St. Louis, MO, USA), and mice were anesthetized with ketamine (80 mg/kg) (Ketavet, Pfizer, Berlin, Germany) as described previously [24]. The murine macrophage cell line J774A.1 and the human epithelial cell line HeLa cells were obtained from China Infrastructure of Cell Lines Resource (China). The cells were cultured in DMEM medium (Gibco, Carlsbad, CA, USA) supplemented with 10% (v/v) FBS (Gibco, Carlsbad, CA, USA) at 37°C with 5% CO_2_.

### Genome sequencing and comparative analysis

The genomic DNA of MB1 was extracted using the FastPure® Blood/Cell/Tissue/Bacteria DNA Isolation Mini Kit (Vazyme Biotech Co.,ltd) according to the manufacturer’s instruction. The purity and quality of the total DNA were verified by agarose gel electrophoresis. Genome sequencing was conducted by Novogene Bioinformatics Technology Co., Ltd. (Beijing, China) using the PacBio Sequel platform and Illumina NovaSeq PE150. The SMRT portal assembly software was used to filter out the low-quality reads and complete the preliminary assembly. Based on the genome, the putative coding genes were retrieved using the GeneMarkS (Version 4.17) (http://topaz.gatech.edu/GeneMark/) software. The tRNAs, rRNAs, and sRNAs were predicted with tRNAscan-SE [25], rRNAmmer [26], and BLAST against the Rfam database [27], respectively. Genomic islands were predicted with IslandPath-DIOMB. PHAST was used for the prophage prediction (http://phast.wishartlab.com/). Seven databases were used for whole genome Blast search to predict gene function, including GO (Gene Ontology), KEGG (Kyoto Encyclopaedia of Genes and Genomes), COG (Clusters of Orthologous Groups), NR (Non-Redundant Protein Database databases), TCDB (Transporter Classification Database), Pfam, and Swiss-Prot. A whole genome Blast search was performed against Virulence Factors of Pathogenic Bacteria database (VFDB) [28]. Based on the assembled genome sequence, combining with the predicted results of the coding genes, the genome maps were generated by the Circos software [29]. Genomic alignment between the MB1 and ATCC 14579 was performed using the MUMmer and LASTZ tools. Genomic synteny was analysed based on the alignment results. Core genes and specific genes were screened using the CD-HIT rapid clustering of similar proteins software with 0.7 length difference cut-off in amino acid and a threshold of 50% pairwise identity. For virulence gene comparison, the proteins encoded by the 10 plasmid-borne virulence genes of MB1 were searched against the proteins in other *Bacillus* via BLASTP, with Evalue of 1e-5, and the uniqueness was assessed based on the percentage of identity from the all-against-all comparison. The GTDBTk-1.4.0 ‘classify_wf’ command was applied to call 120 aligned single-copy bacterial marker protein sequences from the genomes of *Bacillus* sp [30]. The phylogenetic tree was subsequently inferred from the aligned protein sequences using maximum likelihood with substitution model as WAG+G via FastTree [31].

### In vivo infection

MB1 was cultured in 2216E medium to an OD_600_ of 0.8. The bacterial cells were collected after centrifugation and resuspended in PBS to different concentrations. Median lethal dose (LD_50_) was determined as reported previously [23]. For infection of fish, turbots (n = 10) were injected intramuscularly with 100 μl MB1 suspension (5 × 10^5^ CFU/g), 100 μl MB1 spore suspension (5 × 10^5^ CFU/g), or 100 μl PBS (control). For infection of mice, mice (n = 6) were injected intraperitoneally with 100 μl MB1 suspension (2 × 10^6^ CFU/g) or PBS. The liver, kidney, and spleen were aseptically removed from the fish at 12, 24, and 48 h post inoculation (hpi) or from the mice at 3 hpi. The tissues were homogenized in PBS, and the homogenates were serially diluted and plated in triplicate on 2216EA plates. The plates were incubated at 28 °C for 24 h, and the colonies on the plates were counted.

### In vitro infection

J774A.1 cells (10^6^ cells/well) or HeLa cells (10^5^ cells/well) cultured in serum-free Opti-MEM medium (Thermo Fisher, NYC, USA) in 24-well plates were infected with MB1 or MB1 spores at a multiplicity of infection (MOI) of 0 to 10. Alternatively, the cells were treated with 0% to 100% (v/v) of the cell-free culture supernatant of MB1. Cytoplasmic lactate dehydrogenase (LDH) release was evaluated using the CytoTox 96® Non-Radioactive Cytotoxicity Assay kit. The morphological change of the cells was observed using microscope and SEM. Annexin V-FITC/PI staining was performed using the Annexin V-FITC Apoptosis Detection Kit (Solarbio Science, Beijing, China) according to the manufacturer’s instruction. Cytokine release from J774A.1 cells was determined using the QuantiCyto® Mouse IL-1β ELISA kit and the QuantiCyto® Mouse IL-18 ELISA kit (Neobioscience Technology Co, Ltd) according to the manufacturer’s instructions. Treatment of J774A.1 cells with *B. cereus* ATCC 14579 or its culture supernatant was performed as above.

### Immunoblotting analysis

J774A.1 cells were infected with MB1, MB1 spores, or the culture supernatant of MB1 as above. The cell culture supernatant and cell lysate were prepared as described previously [32]. In short, trichloroacetic acid was added to the cell culture supernatant to a final concentration 10% (v/v). The mixture was incubated on ice for 1 h and then centrifuge at 20,000 g for 30 min to obtain. The protein precipitate was washed twice with pre-cooled acetone and then air-dried. To obtain cell lysate, RIPA buffer containing protease and phosphatase inhibitors (Beyotime, Shanghai, China) were added to the cells in 24-well plates, and the plates were placed on ice for 10–15 minutes. All samples were mixed with SDS-PAGE buffer and heated at 100 °C for 10 min. The proteins were separated on 12% polyacrylamide gels and transferred onto nitrocellulose membranes (GE Healthcare, USA). The membranes were blocked in 5% skim milk in Tris-buffered saline with Tween-20 (TBST) and incubated overnight with the primary antibody against caspase-1 (1:1000 dilution) or GSDMD (1:1000 dilution) (Abcam, Cambridge, MA, UK). The membranes were then incubated with horseradish peroxidase-conjugated secondary antibody (1:5000) (ABclonal, Wuhan, Hubei, China) for 1 h. The proteins were visualized using BeyoECL Plus kit (Beyotime, Shanghai, China) and imaged with the GelDoc XR System (Bio-Rad, USA).

### Statistical analysis

Statistical analysis was carried out using GraphPad Prism 7.0 (GraphPad, San Diego, CA, USA). Statistical significance was determined with Student’s *t* test for two groups. p < 0.05 was considered statistically significant.

## Results

### Biological and phylogenetic characteristics of B. cereus MB1

**Figure 1.**
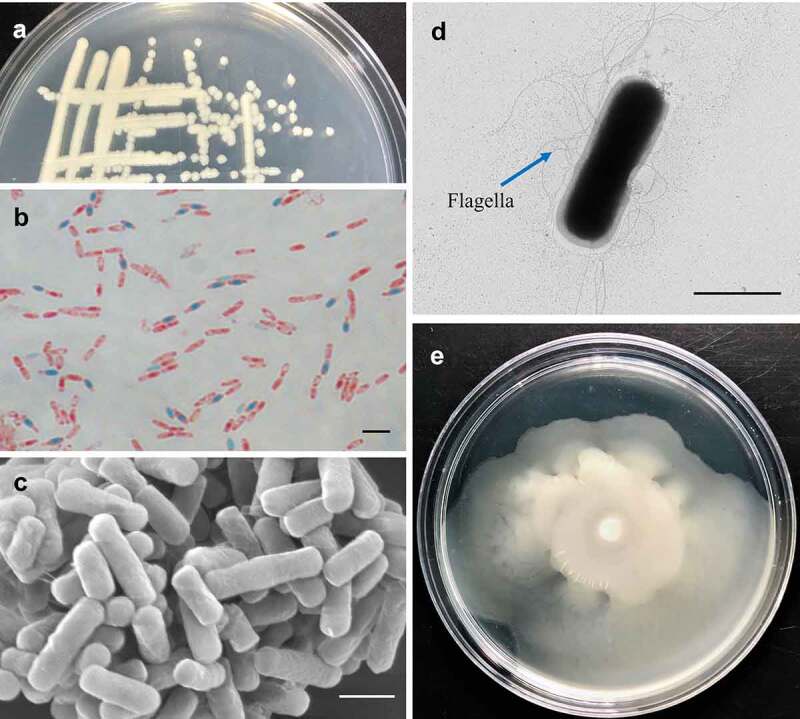
Biological characteristics of *Bacillus cereus* MB1. (**a**) MB1 colonies on Marine 2216EA plate. (**b**) MB1 spores stained with malachite green and safranine. (**c** and **d**) MB1 observed with scanning (**c**) and transmission (**d**) electron microscopes. (**e**) Growth of MB1 on marine 2216E containing 0.3 (w/v) agar. For panels b, c, and d, the scale bar is 2 μm.

Strain MB1 was isolated from the Challenger Deep of the Mariana Trench (Figure S1). MB1 formed rough and milky white colonies on marine 2216E medium after 24 h growth at 28°C (Figure 1a). It possesses peripheral flagella and can produce endospores that are oval-shaped and located centrally in the cell (Figure 1(b–d)). MB1 grew at 16–42°C, pH 5–11, and 0 to 6% NaCl (Figure S2), and exhibited motility in 0.3% agar (Figure 1e). MB1 was preliminarily classified into the *Bacillus* group based on the16S rRNA gene sequence, which showed that MB1 was closely related to *B. cereus* in phylogeny (Figure S3). Genome analysis revealed that the average nucleotide identity (ANI) between MB1 and *B. cereus* ATCC 14579 (type strain) is 98.93%, which is higher than the species demarcation threshold (95%), indicating that MB1 is a *B. cereus*. In support of this, when all the genes of MB1 were screened in the Non-Redundant Protein Database (NR), most of the matches were found in *B. cereus* (Figure S3).

### Genome features of MB1

**Figure 2.**
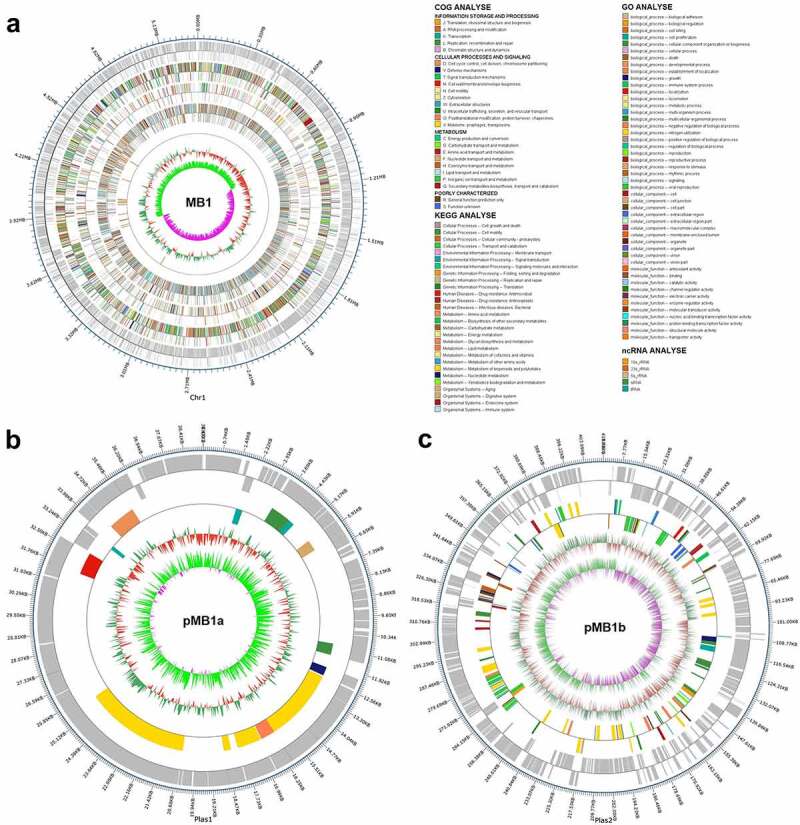
Circular maps of *Bacillus cereus* MB1. Circular maps of MB1 chromosome (**a**), pMb1a (**b**), and pMb1b (**c**) are shown. The scales on the outside circles indicate the coordinates of the genome/plasmids. The circles from outside to inside represent the following: COG annotated genes, KEGG annotated genes, GO annotated genes, ncRNA, GC content, and GC skew. Gray represents unannotated genes.

The general features of the genome of MB1 are shown in Table 1 and Figure 2. The complete genome of MB1 consists of a circular chromosome of 5,327,038 base pairs (bp) and two circular plasmids (named pMB1a and pMB1b) of 39154 bp and 411764 bp, respectively. The average GC contents of the circular chromosome, pMB1a, and pMB1b are 35.36%, 35.35%, and 33.44%, respectively. MB1 contains 5966 genes, which account for 84.0 % of the total genome. Of these genes, 5493 are encoded in the chromosome, and 57 and 416 are encoded in pMB1a and pMB1b, respectively. All non-coding RNAs are located on the chromosome, including 14 operons of 23S, 5S, and 16S ribosomal RNA, 106 tRNA, and 4 sRNA. MB1 possesses 12 genomic islands (GIs), one of which is located on the plasmid (pMB1b). The number of genes in these GIs ranges from 7 to 47 (Table S1). MB1 also possesses 13 prophages distributed on the chromosome and plasmids (Table S2), and 7 CRISPR on the chromosome.

**Table 1.** The general genome features of *Bacillus cereus* MB1 in comparison with that of ATCC 14579.

Category	MB1				ATCC 14579
	Chromosome	pMB1a	pMB1b	Total	Genome
Genome Size (bp)	5,327,038	39154	411764	5,777,956	5,426,909
Gene Number	5493	57	416	5966	5366
Gene Length	4499105	36183	318472	4,853,760	4,559,996
GC Content	35.36	35.35	33.44	35.88	35.3
% of Genome (Genes)	84.45	92.41	77.34	84.0	84.0
tRNA	106	0	0	106	unknown
rRNA (16S-23S-5S)	14	0	0	14	13
sRNA	4	0	0	4	10
Prophage	10	1	2	13	6
Virulence Genes	292	0	10	302	138

### Comparative analysis of the genomes of MB1 and B. cereus ATCC 14579

**Figure 3.**
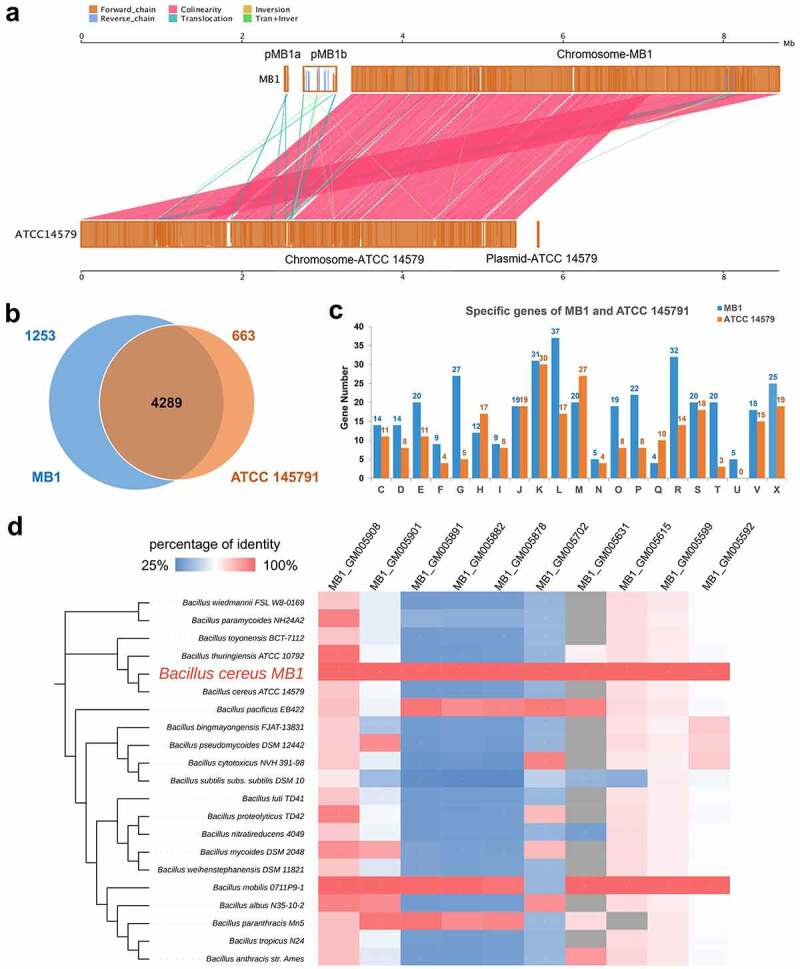
Comparative genomic analysis of MB1 and ATCC 14579. (**a**) Alignment of the genomes of MB1 and ATCC 14579. (**b**) a Venn diagram showing the shared and unique genes of MB1 and ATCC 14579. (**c**) the numbers of MB1- and ATCC 14579-specific genes assigned to the COG categories. (**d**) the comparison of the 10 plasmid-encoded virulence genes of MB1 with their closest homologues in other *Bacillus*. Grey represents absence of the gene in the genome. Color transition from blue to red represents increasing percentage of identity from 25% to 100%.

Comparative genomic analysis was performed with MB1 and *B. cereus* ATCC 14579, which is an opportunistic pathogen [33,34] and, as shown above, is most closely related to MB1. As shown in Table 1, the genome size, GC content, gene number, and percentage of genes are comparable between MB1 and ATCC 14579. There is a strong collinearity between the two genomes, however, many genomic translocations and inversions occur in MB1, especially in the plasmids (pMB1a and pMB1b) (Figure 3a). MB1 and ATCC 14579 share 4,289 genes, and encodes 1,253 and 663 specific genes, respectively (Figure 3b). The MB1 specific genes mainly correspond to the COG categories of R (general function prediction only), L (replication, recombination and repair), S (function unknown), C (energy production and conversion), J (translation, ribosomal structure and biogenesis), M (cell wall/membrane/envelope biogenesis), N (cell motility), O (posttranslational modification, protein turnover, chaperones), and X (mobilome: prophages, transposons) (Figure 3c).

### Virulence genes in the MB1 genome

Using the VFDB database as a reference, 302 genes related to virulence in the MB1 genome were annotated (Table S3). Of these genes, 292 are on the chromosome and 10 are on pMB1b. For the 10 virulence genes on pMB1b, most of them lack close counterparts in the genomes of terrestrial *Bacillus* sp., including ATCC 14579. High-similarity homologues of these virulence genes were only found in *Bacillus mobilis* 0711P9–1, *Bacillus paranthracis* Mn5, and *Bacillus pacificus* EB422 (Figure 3d), which are from ocean sediment or seawater. The virulence genes of MB1 are involved in diverse functions. For example, the genes *fleQ*, *lafK*, *flmH*, *fliR*, *fliG*, *CheR*, and *fliH* are involved in flagella synthesis, assembly, and movement. Several genes encoding typical pore-forming toxins associated with foodborne illness were identified, including *hblB*, *hblC*, and *hblD*, which encode haemolysin BL (HBL), *nheA*, *nheB*, and *nheC*, which encode the non-haemolytic enterotoxin (NHE), and *cytK*, which encodes cytotoxin K (CytK). Genes encoding proteins/enzymes contributing to haemolysis (*hlyIII*, haemolysin type III) and immune escape (*inhA*, immune inhibitor A peptidases in family M6) were also identified. Other putative virulence genes include *BCQ_0745*, *plcA*, and *piplc*, which are involved in the synthesis of sphingomyelinase (SMase), phosphatidylinositol-specific phospholipase C (PI-PLC), and phosphatidylcholine-specific phospholipase C (PC-PLC), respectively, and genes coding for cytotoxicity-associated proteins, such as Cereolysin O, Aureolysin, *M. catarrhalis* adherence protein (McaP), and Listeria adhesion protein.

### MB1 causes lethal infection in fish and mice

**Figure 4.**
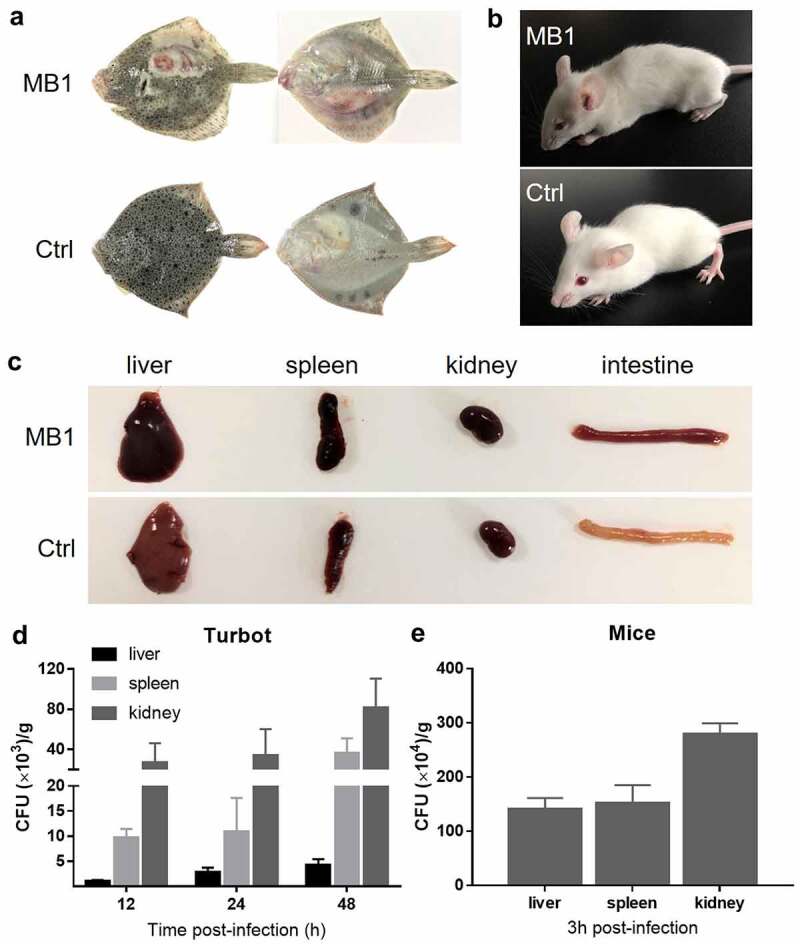
Pathogenicity of MB1 to turbot and mice. (**a**, **b**) Turbot (**a**) and mice (**b**) were infected with or without (Ctrl) MB1 for 24 h and 2 h, respectively, and observed for clinical symptoms. (**c**) Tissues from MB1-infected moribund mice and uninfected mice were examined. (**d**, **e**) Turbot (**d**) and mice (**e**) were inoculated with MB1, and bacterial numbers (shown as colony forming unit, CFU) in the liver, spleen, and kidney were determined at 12, 24, and 28 h (turbot) or 3 h (mice) post-infection. The results are the means of triplicate experiments and shown as means ± SD.

The pathogenicity of MB1 was evaluated in fish and mice infection models. The LD_50_ of MB1 to fish (turbot *Scophthalmus maximus*) and mice were 1 × 10^5^ CFU/g and 5 × 10^5^CFU/g, respectively. Turbot infected with MB1 at the dose of 5 × 10^5^CFU/g developed skin ulcers and haemorrhage at the injection site at 24 h post infection (hpi) (Figure 4a). Mice infected with MB1 at the dose of 2 × 10^6^CFU/g became listlessness at 2 hpi, and died with convulsion at about 3 hpi (Figure 4b). Autopsy showed that the liver, spleen, kidney, and intestine of the infected animal were congested and swollen (Figure 4c). Following intramuscular injection, MB1 rapidly disseminated into the liver, spleen, and kidney of turbot in a tissue- and time-dependent manner, with relatively the highest bacterial recoveries from kidney (Figure 4d). Similar observations were made with the mice model of infection (Figure 4e).

### Both MB1 and its culture supernatant are cytotoxic to mammalian cells

**Figure 5.**
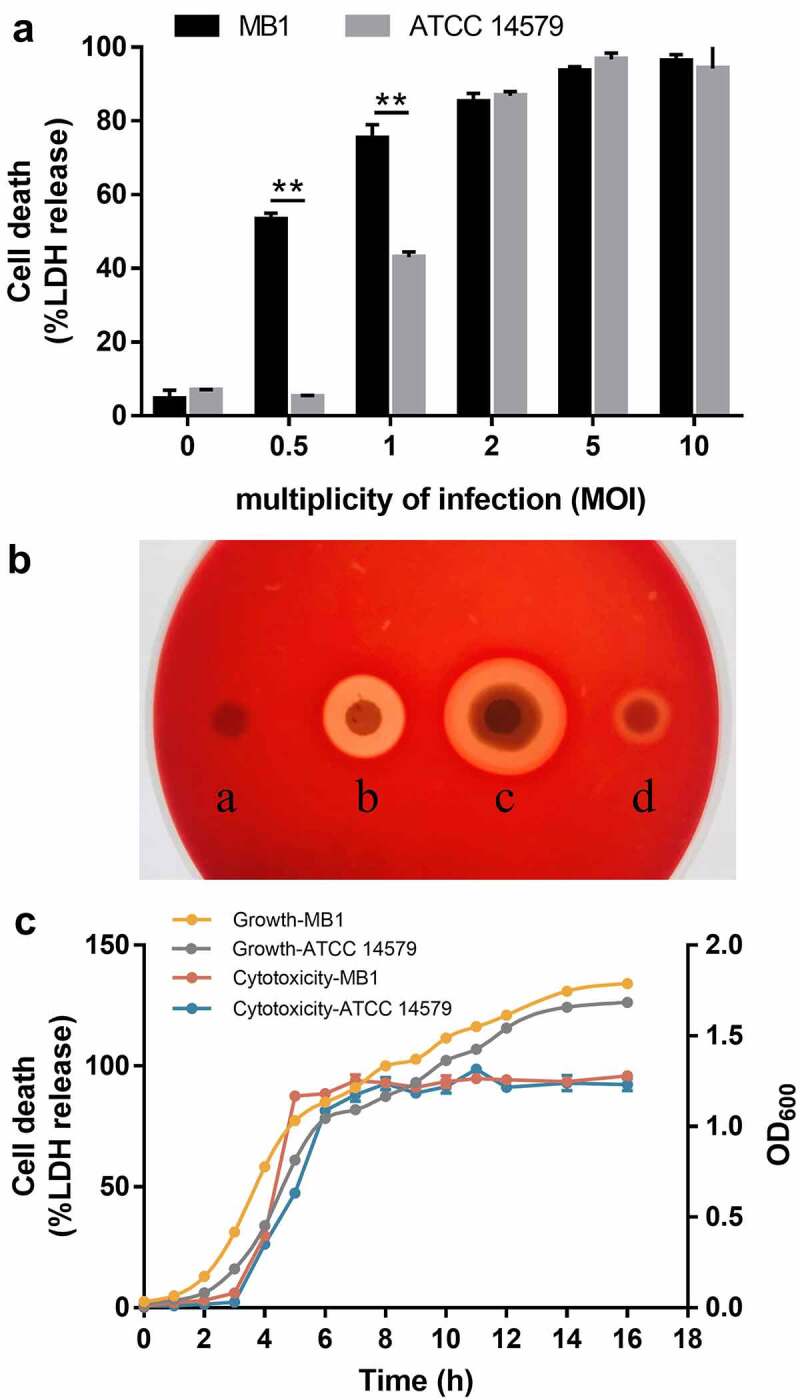
Cytotoxicity of MB1. (**a**) J774A.1 cells were incubated with MB1 or Bacillus cereus ATCC 14579 for 2 h at a multiplicity of infection (MOI) of 0 to 10, and then measured for lactate dehydrogenase (LDH) release. Data are the means of triplicate experiments and shown as means ± SD. ** p < 0.01 (Student’s t test). (**b**) Hemolytic activity of MB1. a, PBS (control); b, 2% TritonX-100; c, MB1 suspension; d, MB1 supernatant. (**c**) the supernatants of MB1 and ATCC 14579 were collected at different times of culturing and incubated with J774A.1 (5% v/v) for 2 h. LDH release was then measured. Data are the means of triplicate experiments and shown as means ± SD.

Since MB1 possesses several toxin genes, we evaluated its cytotoxic potential. When incubated with the mouse macrophages J774A.1 cells for 2 h, MB1 induced 50% and 100% cell lysis at the MOI of 0.5 and 5, respectively, as measured by LDH release (Figure 5a). At the lower MOI (0.5 and 1), MB1 induced significantly higher amounts of cell death than ATCC 14579. In agreement with the observed cytolytic effect of MB1, both the bacterial cells and the culture supernatant of MB1 displayed haemolytic activity (Figure 5b). To examine the expression kinetics of the toxic factors in MB1, the supernatants of MB1 as well as ATCC 14579 were collected at different growth stages and tested for cytotoxicity. The result showed that for both strains, the degree of cytotoxicity paralleled roughly the growth of the bacteria during the early logarithmic phase and reached maximum around the mid-logarithmic phase when the OD_600_ was about 1 (Figure 5c).

### MB1 and its culture supernatant trigger pyroptosis in a dose-dependent manner

**Figure 6.**
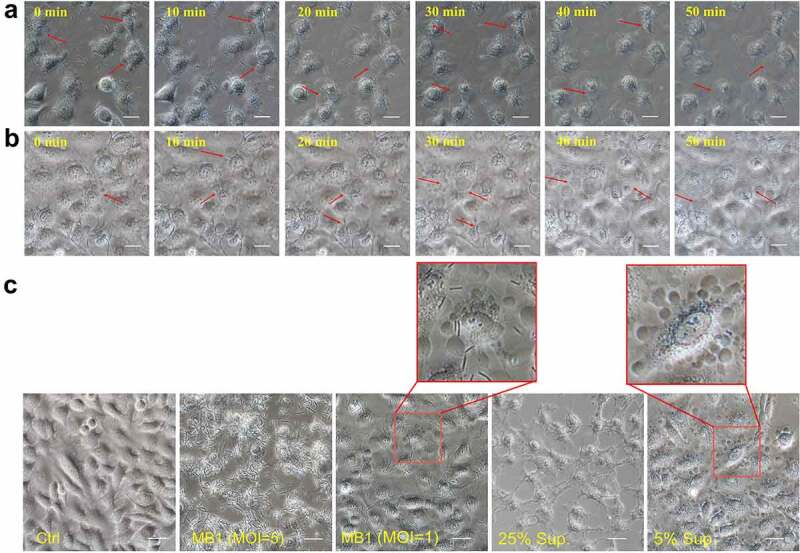
Cell death induced by MB1 and its culture supernatant at different doses. (**a**, **b**) Time-lapse images of HeLa cells treated with MB1 (MOI = 1) (A) or its supernatant (5%) (B). Red arrows indicate bubbled cells. (**c**) HeLa cells were incubated with or without (Ctrl) MB1 (MOI = 1 or 5) or MB1 supernatant (sup.) (5% or 25%) for 2 h and observed with a microscope. For panels a to c, the scale bar is 30 μm.

**Figure 7.**
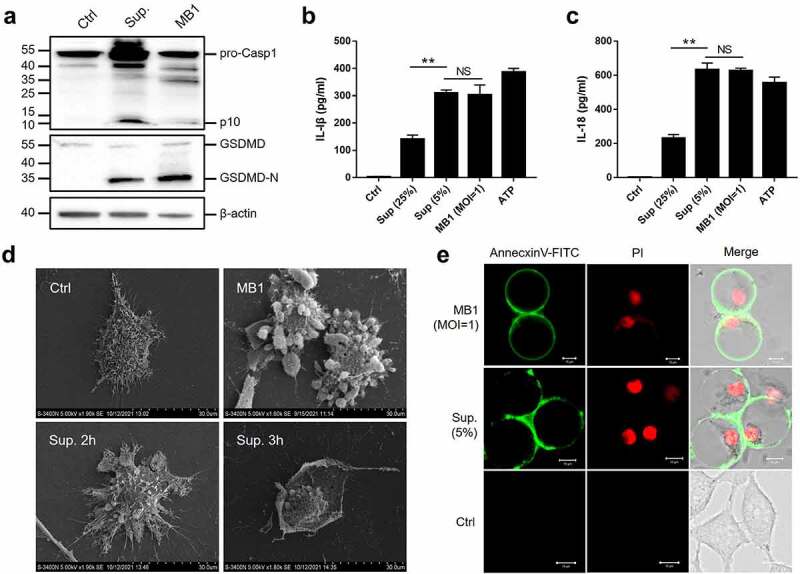
MB1 and its culture supernatant induce pyroptosis at low doses. (**a**) J774A.1 cells were primed with LPS and infected with or without (Ctrl) MB1 (MOI = 1) or the supernatant (Sup.) (5% v/v) for 2 h. The cell lysate and culture supernatant were immunoblotted with antibodies against caspase-1 (Casp1), GSDMD, or β-actin (loading control). (**b**, **c**) LPS-primed J774A.1 cells were infected with or without (Ctrl) MB1 or the supernatant (5% or 25%) or treated with ATP for 2 h, and the release of IL-1β (B) and IL-18 (c) was measured. Values are shown as means ± sd (N = 3). N, the number of replicates. ** p < 0.01 (Student’s *t* test). NS, no significance. (**d**) LPS-primed J774A.1 cells were infected with or without (Ctrl) MB1 or the supernatant (5%) for 2 h or 3 h, and then observed with a scanning electron microscope. (**e**) LPS-primed HeLa cells were infected with or without (Ctrl) MB1 or the supernatant (5%) for 3 h. The cells were stained with Annexin V-FITC/PI and observed with a confocal microscope.

Microscopy showed that at the low dose, MB1 (MOI = 1) and its supernatant (5%) induced a gradual cell death, during which the cells underwent a process of swelling up, while at the high dose, MB1 (MOI = 5) and the supernatant (25%) caused rapid rupture of the target cells (Figure 6). At low dose, both MB1 and the supernatant treatments led to activation of caspase-1, cleavage of gasdermin D (GSDMD), and secretion of IL-1β and IL-18 (Figure 7(a–c)), implying activation of pyroptosis. At the high concentration (25%), the supernatant induced significantly less release of IL-1β and IL-18 (Figure 7(b,c)). Microscopy showed that the cells treated with MB1 or the supernatant at low dose exhibited characteristics of pyroptosis, including the appearance of cellular protrusion, loss of membrane integrity, and a centralized nucleus in a deflated cell body (Figure 7d). The swollen cells before rupture were double stained by Annexin V-FITC and propidium iodide (PI) (Figure 7e).

### The pathogenicity of MB1 is severely attenuated during sporulation and fully regained after spore germination

**Figure 8.**
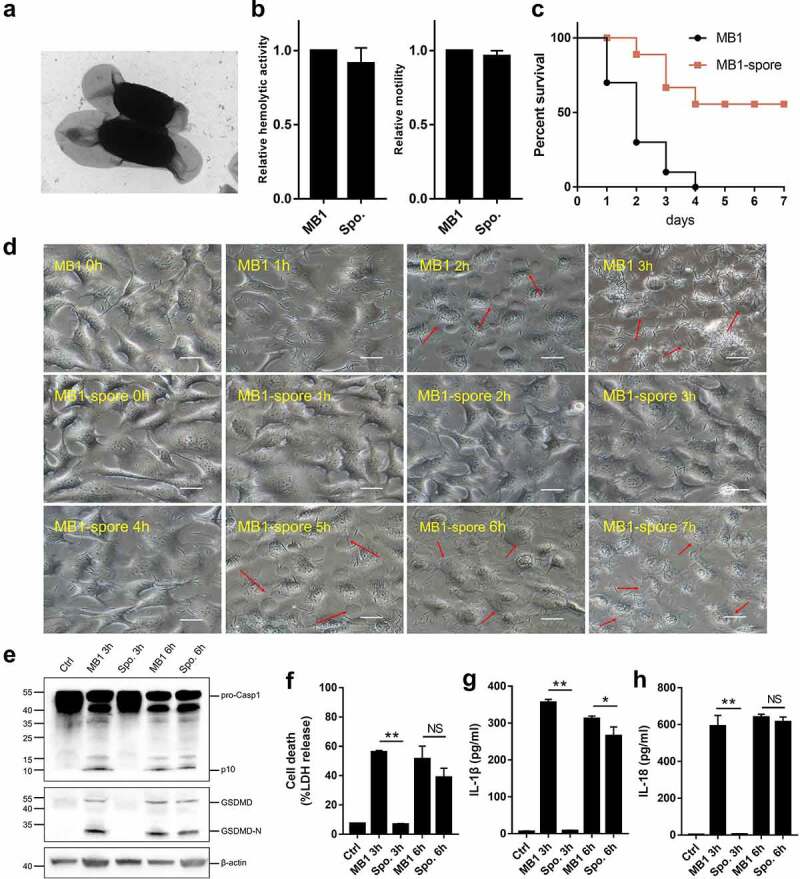
Pathogenicity of MB1 spores. (**a**) MB1 spores observed with a transmission electron microscope. (**b**) the swimming and haemolytic abilities of MB1 spores in comparison with that of MB1. (**c**) Turbots were infected with MB1 or the spores, and the survival of the fish was monitored for 7 days. (**d**) HeLa cells were incubated with MB1 or the spores (MOI = 1) for different hours and observed with a microscope. Red arrows indicate pyroptotic cells. The scale bar is 30 μm. (e – h) J774A.1 cells were infected with or without (Ctrl) MB1 or the spores (MOI = 1) for 3 or 6 h. The cell lysate and culture supernatant were mixed and immunoblotted with antibodies against caspase-1 (Casp1), GSDMD, or β-actin (loading control) (**e**). Release of lactate dehydrogenase (LDH) (**f**), IL-1β (**g**), and IL-18 (**h**) was measured. For panels b, f, g, and h, values are shown as means ± sd (N = 3). N, the number of replicates. ** p < 0.01, * p < 0.05 (Student’s *t* test). NS, no significance.

Since, as shown above, MB1 was able to form spores, we examined whether sporogenesis affected the pathogenicity of MB1. For this purpose, MB1 was induced to reach over 95% of sporulation rate, and the spores were confirmed microscopically (Figure 8a). Compared to MB1, the spores showed similar haemolytic activity and swimming ability (Figure 8b). However, when inoculated into turbot, the spores induced a much lower mortality rate than MB1 (Figure 8c). When incubated with HeLa cells in the serum-free Opti-MEM medium, the spores caused no visible cellular damage in the first 3 h. After 3 h, the spores visibly reverted back to the vegetative growth status, as their shapes changed from the coccus-form into the rod-form (Figure 8d). Cell death induced by the resuscitated spores was evident at and after 5 h. Similarly, when incubated with the macrophages J774A.1, the spores induced apparent activation of caspase-1 and GSDMD and release of IL-1β/IL-18 only after resuscitation (Figure 8(e–h)).

## Discussion

In this study, we reported the characterization of a deep sea *B. cereus*, MB1, isolated under aerobic condition, which is in agreement with the fact that the dissolved oxygen (3.43 mL/L) in Mariana Trench is sufficient to support aerobic organisms [35]. MB1 was classified as a member of the *B. cereus* group based on the 16S rRNA gene sequence and the ANI value. *B. cereus*, as well as *B. subtilis*, *B. toyonensis*, and *B. wiedmannii*, have been identified in deep sea hydrothermal vents or cold seeps [23,24,36], suggesting a wide distribution of *Bacillus* in the deep sea environment. Genome sequencing revealed that MB1 contains a circular chromosome and two plasmids. One of the plasmids carries a large number (416) of genes, which differ apparently in GC content from the genes of the chromosome, indicating different sources of origin for the plasmid and chromosomal genes. MB1 also harbours 13 prophages in the chromosome as well as the plasmids. These genetic features suggest that MB1 may have acquired new genetic traits or exchanged DNA information with the environment via mobile genetic elements.

Genome analysis indicated that compared with ATCC 14579, MB1 contains many translocations, inversions and insertions, especially in the additional pMB1b plasmid. This unique plasmid harbours a number of virulence genes, whose close counterparts exist only in a very few bacillus species, which, interestingly, were from the Pacific or Indian Ocean. These results suggest that these genes may have emerged under the selection of the marine environments. It has been reported that phages in marine environments mediate gene transfer between hosts through evolution [37]. In MB1, the number of prophage was found to be more than twice that in ATCC 14579, implying that phage infection may have promoted heritable horizontal gene transfer and gene reshuffling in MB1. For organisms living under extreme conditions, such as the Mariana Trench where the water pressure is around 100 MPa and the temperature is 1.67–2.40 °C [35], special adaptation mechanisms have to be developed. In our study, we found that MB1-specific genes were mainly grouped to the R, L, S, C, J, M, N, O, and X categories. This observation is in line with the previous reports that in deep-sea bacteria, the genes of the C, M, and N categories may be associated with energy production/conservation and structural resistance against high hydrostatic pressure (HHP) [37,38], and the genes of the L, J, and O categories may be involved in overall gene expression to compensate for the loss of biological activity under HHP [39,40]. The flagella systems of MB1 are likely essential for bacterial motility and living at low temperature as reported previously for other bacterial strains [41].

B.*cereus* is a well-known causative agent of foodborne disease. The pathogenicity of *B. cereus* is closely related to its ability to produce toxins. Two three-component enterotoxins, i.e. HBL and NHE, and one single-component toxin, i.e. CytK, are considered the main toxicity factors causing diarrhoeal food poisoning [9,42]. Significantly, the ability to produce enterotoxins varies considerably among different strains of *B. cereus*, and one strain may produce all or part of HBL, NHE, and CytK [9,43,44]. In our study, MB1 was found to harbour more than 300 annotated virulence genes, including the HBL/NHE- and the CytK-encoding gene clusters, implying a high potential of pathogenicity. Previous studies showed that besides enterotoxins, haemolysins are also a key feature of *B. cereus* [45]. Although HBL, NHE, and CytK can induce erythrocyte lysis owing to their pore-forming ability, *B. cereus* still expresses haemolysins (I, II, and III) to execute haemolysis. MB1 lacks the haemolysin II gene but has the haemolysin I and III genes, suggesting a haemolytic ability, which was confirmed by the observation that MB1 effective lysed mammalian red blood cells. In addition to toxin-related genes, MB1 also contains the genes encoding phospholipases, including SMase, PI-PLC, and PC-PLC. Phospholipases are known to be toxicity factors for a variety of pathogens, and can promote haemolysis and tissue damage by inducing the degranulation of human neutrophils [46–48]. Together, these results indicated a genetic predisposition for MB1 to be a pathogen.

In line with the presence of a large group of virulence associated genes in the genome of MB1, infection studies revealed that MB1 was pathogenic to fish and mice and induced rapid mortality of the host after intraperitoneal or intramuscular injection. Following inoculation, *B. cereus* was soon recovered from the internal organs distant from the infection site, indicating a strong tissue dissemination capacity. The infected animals exhibited marked clinical signs, including tissue congestion and haemorrhage, which may be due to the action of the enterotoxins and haemolysins. Cellular study showed that both the bacterial cells and the culture supernatant of MB1 were highly toxic to mammalian cells, suggesting that the cytotoxins were produced extracellularly by MB1. In support of this observation, the cytotoxic activity of MB1 was highest when the bacterial growth reached the mid-logarithmic stage. Hence, the production of the cytotoxins was dynamically regulated during the growth of MB1. Compared to ATCC 14579, MB1 exhibited significantly stronger cytolytic activities at lower MOI, indicating that at lower infection doses, MB1 was more virulent than ATCC 14579, which may be accounted for by the presence of the much more virulence genes in MB1.

Pyroptosis is a form of programmed cell death executed by the gasdermin family proteins. Pyroptotic cells exhibit lytic cell death and activation of pro-inflammatory cytokines like IL-1β and IL-18 [49,50]. Pyroptosis can be induced by multiple stimuli, including bacterial toxins and pathogens [51,52]. In our study, we found that MB1-infected cells displayed typical characteristics of pyroptosis, including permeabilization of the plasma membrane, swelling of the cells, Casp1 activation, GSDMD cleavage, and release of IL-1β and IL-18. Previous reports showed that NHE or HBL released by *B. cereus* could solely induce pyroptosis by activating the NLRP3 inflammasome [13,15]. Hence, it is possible that the enterotoxins and haemolysins of MB1 may be the actual and direct driver for the induction of pyroptosis. Interestingly, we observed that the cells infected with MB1 were stained by Annexin V/PI, which is a common method for detecting apoptosis [53]. It is possible that, as reported previously [50], during the process of cell death the early membrane rupture exposed the inner leaflet of the plasma membrane to the extracellular surface, so that the phospholipid phosphatidylserine became accessible to annexin V.

Spores are a unique strategy for bacteria to overcome challenging environmental conditions such as heating, freezing, and drying [54,55]. Although spores are metabolically dormant, they are the infection ways for some spore-forming pathogens [53,56]. After contacting the host, the spores are induced to germinate and grow into metabolically active cells, which then colonize the host and secrete toxins to exert pathogenicity [56–58]. In our study, MB1 could be induced into sporulation and exist predominantly as spores. The spores retained the haemolytic and swimming ability, but were severely impaired in infectivity and cytotoxicity. It is known that the dormant spores need specific stimulating signals, generally low-molecular-weight biomolecules in a favourable environment, to return to vegetative growth [59,60]. In the case of MB1, the spores were able to transit into the vegetative growth state in the mammalian cell culture medium and regain fully the pyroptosis-inducing capacity. This observation raises a concern that some *Bacillus*, such as MB1, may be innocuous in the deep sea environment but become toxic once being brought into a land environment suitable for germination.

In conclusion, we demonstrated in this study that a *B. cereus* isolate, MB1, from the Mariana Trench is cytotoxic and can induce lethal infections in fish and mice. The pathogenicity of MB1 is dramatically reduced during sporulation but is fully restored when the spores resuscitate. These results promote our understanding of the genetics and virulence mechanism of deep-sea *B. cereus*.

## Supplementary Material

Supplemental Material

## Data Availability

The datasets generated for this study can be found in the GenBank accession numbers CP091971, CP091972 and CP091973.
