# Supplementary material for: Characterization of the pathogenicity of a Bacillus cereus isolate from the Mariana Trench
Source: Virulence. 2022 Jun 22;13(1):1062–75. doi: 10.1080/21505594.2022.2088641 (PMC9235904; doi:10.1080/21505594.2022.2088641)
Supplement: Supplemental Material [file KVIR_A_2088641_SM1605.docx]

**Supplemental data**

**Figure S1.** The sampling site in the Challenger Deep. The map was created with MATLAB 2018b. The gridded elevation data from ETOPO5 are adopted for the bottom topography and the coastline. The sampling site in the Challenger Deep is marked by the yellow star.


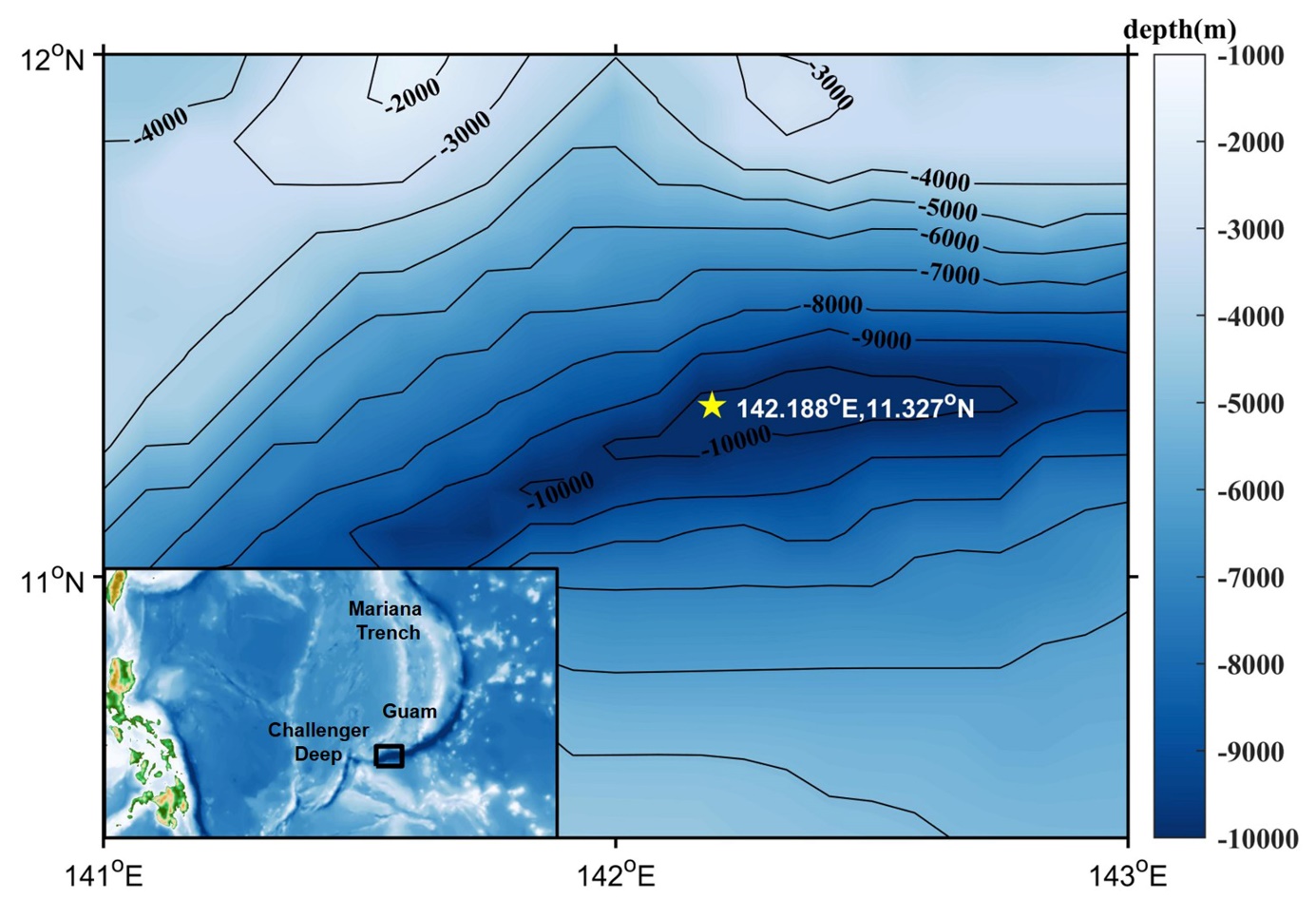


**Figure S2**. Growth of *Bacillus cereus* MB1 at different temperatures (A), pH (B) and NaCl concentrations (C). MB1 was cultured in marine 2216E medium, and bacterial growth was determined by measuring cell density at various time points. The results are the means of triplicate experiment and shown as means ± SD.


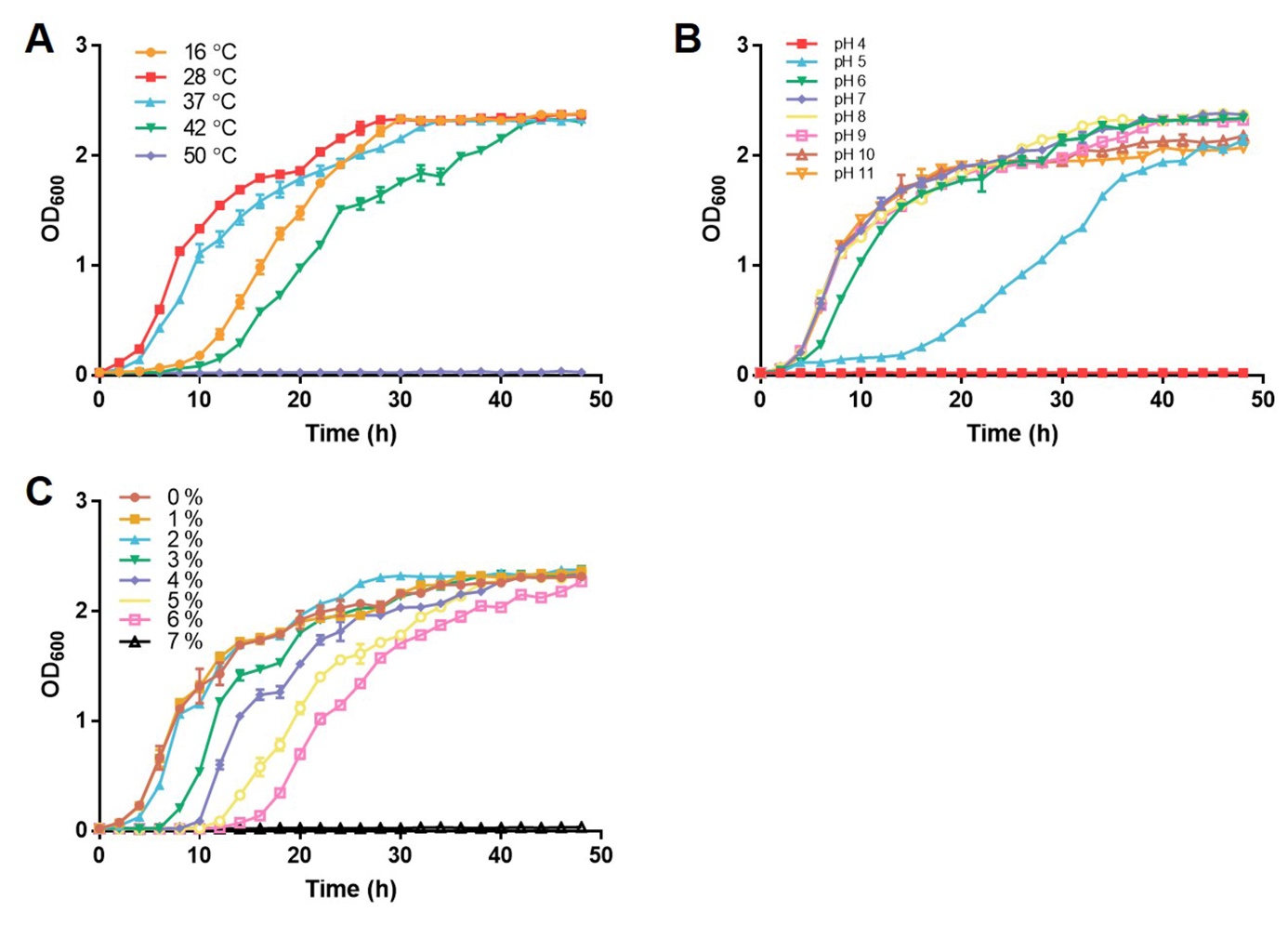


**Figure S3.** Phylogenetic analysis of *Bacillus cereus* MB1. (A) The neighbor-joining (NJ) tree was based on the 16S rRNA gene sequences of MB1 and the type strains of *Bacillus* group. Numbers beside the internal branches indicate bootstrap values based on 1000 replications. (B) The genes of MB1 were searched against the NR database, and the numbers of matched genes in the Top 20 species are shown.

**
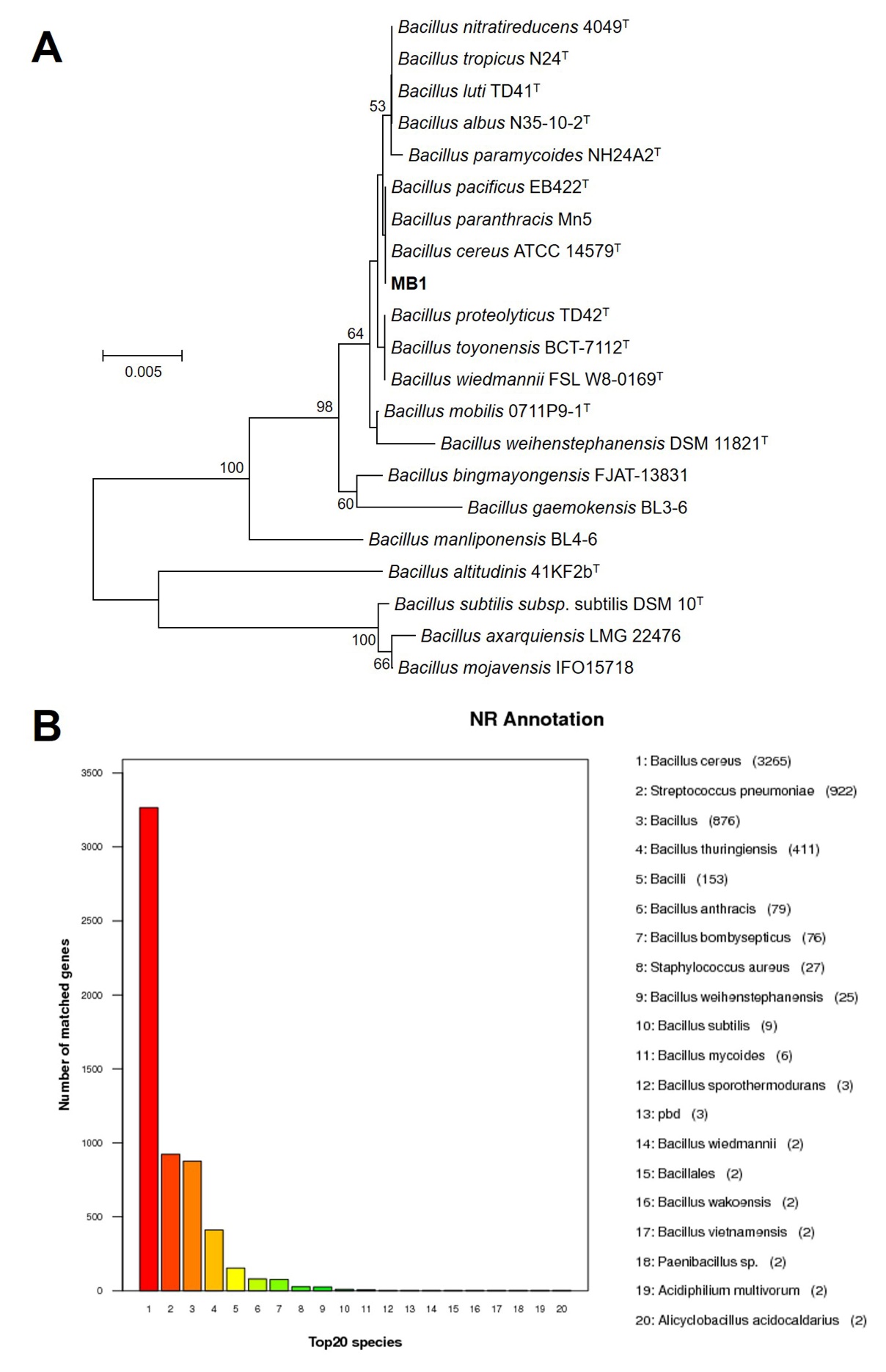
**

**Table S1.** Genomic islands in the genome of MB1

| GI ID | Location | Start | End | Length (bp) | GC% | Gene ID |
| --- | --- | --- | --- | --- | --- | --- |
| GIs001 | Chr | 489601 | 494491 | 4891 | 33.61 | M1_GM000571, M1_GM000572,  M1_GM000573, M1_GM000574,  M1_GM000575, M1_GM000576,  M1_GM000577, M1_GM000578,  M1_GM000579, M1_GM000580 |
| GIs002 | Chr | 620574 | 627282 | 6709 | 29.97 | M1_GM000705, M1_GM000706,  M1_GM000707, M1_GM000708,  M1_GM000709, M1_GM000710,  M1_GM000711, M1_GM000712,  M1_GM000713, M1_GM000714,  M1_GM000715, M1_GM000716,  M1_GM000717 |
| GIs003 | Chr | 1702213 | 1714258 | 12046 | 33.25 | M1_GM001820, M1_GM001821,  M1_GM001822, M1_GM001823,  M1_GM001824, M1_GM001825,  M1_GM001826, M1_GM001827,  M1_GM001828, M1_GM001829,  M1_GM001830, M1_GM001831,  M1_GM001832, M1_GM001833 |
| GIs004 | Chr | 1972971 | 1984455 | 11485 | 40.52 | M1_GM002101, M1_GM002102,  M1_GM002103, M1_GM002104,  M1_GM002105, M1_GM002106,  M1_GM002107, M1_GM002108,  M1_GM002109, M1_GM002110 |
| GIs005 | Chr | 2119369 | 2127321 | 7953 | 37.41 | M1_GM002238, M1_GM002239,  M1_GM002240, M1_GM002241,  M1_GM002242, M1_GM002243,  M1_GM002244 |
| GIs006 | Chr | 2214861 | 2224230 | 9370 | 36.36 | M1_GM002332, M1_GM002333,  M1_GM002334, M1_GM002335,  M1_GM002336, M1_GM002337,  M1_GM002338, M1_GM002339,  M1_GM002340 |
| GIs007 | Chr | 2648504 | 2657059 | 8556 | 37.66 | M1_GM002822, M1_GM002823,  M1_GM002824, M1_GM002825,  M1_GM002826, M1_GM002827,  M1_GM002828, M1_GM002829 |
| GIs008 | Chr | 2755808 | 2768274 | 12467 | 33.06 | M1_GM002922, M1_GM002923,  M1_GM002924, M1_GM002925,  M1_GM002926, M1_GM002927,  M1_GM002928, M1_GM002929,  M1_GM002930, M1_GM002931,  M1_GM002932, M1_GM002933,  M1_GM002934, M1_GM002935,  M1_GM002936, M1_GM002937,  M1_GM002938, M1_GM002939 |
| GIs009 | Chr | 3896717 | 3912993 | 16277 | 34.29 | M1_GM004077, M1_GM004078,  M1_GM004079, M1_GM004080,  M1_GM004081, M1_GM004082,  M1_GM004083, M1_GM004084,  M1_GM004085, M1_GM004086,  M1_GM004087, M1_GM004088 |
| GIs010 | Chr | 4661123 | 4681839 | 20717 | 31.11 | M1_GM004795, M1_GM004796,  M1_GM004797, M1_GM004798,  M1_GM004799, M1_GM004800,  M1_GM004801, M1_GM004802,  M1_GM004803, M1_GM004804,  M1_GM004805, M1_GM004806,  M1_GM004807, M1_GM004808,  M1_GM004809, M1_GM004810,  M1_GM004811, M1_GM004812,  M1_GM004813, M1_GM004814,  M1_GM004815, M1_GM004816,  M1_GM004817, M1_GM004818,  M1_GM004819, M1_GM004820,  M1_GM004821, M1_GM004822,  M1_GM004823, M1_GM004824,  M1_GM004825, M1_GM004826,  M1_GM004827, M1_GM004828,  M1_GM004829, M1_GM004830,  M1_GM004831, M1_GM004832,  M1_GM004833, M1_GM004834,  M1_GM004835, M1_GM004836,  M1_GM004837, M1_GM004838,  M1_GM004839, M1_GM004840,  M1_GM004841 |
| GIs011 | Chr | 4798207 | 4810652 | 12446 | 33.38 | M1_GM004972, M1_GM004973,  M1_GM004974, M1_GM004975,  M1_GM004976, M1_GM004977,  M1_GM004978, M1_GM004979,  M1_GM004980, M1_GM004981 |
| GIs012 | pMB1b | 274212 | 280025 | 5814 | 33.54 | M1_GM005829, M1_GM005830,  M1_GM005831, M1_GM005832,  M1_GM005833, M1_GM005834,  M1_GM005835, M1_GM005836 |

**Table S2.** Prophages in the genome of MB1

| Prophage ID | Location | Start | End | Length (bp) | GC% | Gene No. |
| --- | --- | --- | --- | --- | --- | --- |
| Prophage_1 | pMB1a | 1777 | 26416 | 24640 | 36 | 39 |
| Prophage_2 | pMB1b | 309279 | 364432 | 55154 | 31.99 | 64 |
| Prophage_3 | pMB1b | 365961 | 398255 | 32295 | 35.46 | 35 |
| Prophage_4 | Chromosome | 325747 | 361433 | 35687 | 34.03 | 40 |
| Prophage_5 | Chromosome | 486373 | 554931 | 68559 | 34.01 | 69 |
| Prophage_6 | Chromosome | 1454601 | 1522956 | 68356 | 35.05 | 65 |
| Prophage_7 | Chromosome | 1621646 | 1641778 | 20133 | 37.78 | 19 |
| Prophage_8 | Chromosome | 1938400 | 1975897 | 37498 | 35.16 | 44 |
| Prophage_9 | Chromosome | 2743044 | 2793788 | 50745 | 34.96 | 55 |
| Prophage_10 | Chromosome | 3759709 | 3797334 | 37626 | 37.31 | 38 |
| Prophage_11 | Chromosome | 4572560 | 4642620 | 70061 | 35.34 | 62 |
| Prophage_12 | Chromosome | 4735677 | 4850089 | 114413 | 35.3 | 114 |
| Prophage_13 | Chromosome | 4938448 | 4969624 | 31177 | 35.58 | 31 |

**Table S3**. Virulence genes of MB1 predicted by blast search against the Virulence Factors of Pathogenic Bacteria database (VFDB).

| **Gene ID** | **Identity** | **VFDB ID** | **VFDB name** |
| --- | --- | --- | --- |
| M1_GM003771 | 100 | CVF569 | PlcR |
| M1_GM002154 | 100 | CVF570 | Phosphatidylinositol-specific phospholipase C (PI-PLC) |
| M1_GM004561 | 100 | CVF571 | Phosphatidylcholine-preferring phospholipase C (PC-PLC) |
| M1_GM000126 | 100 | AI153 | peritrichous flagella |
| M1_GM000283 | 100 | CVF564 | Nonhemolytic enterotoxin NHE |
| M1_GM001477 | 100 | CVF563 | Hemolytic enterotoxin HBL |
| M1_GM000920 | 100 | AI128 | Bcp pili |
| M1_GM005244 | 99.9 | TX186 | InhA1, InhA2, InhA3 |
| M1_GM002966 | 99.7 | IA028 | petrobactin |
| M1_GM003697 | 99.6 | CVF567 | Polysaccharide capsule |
| M1_GM000132 | 99.6 | AI153 | peritrichous flagella |
| M1_GM000130 | 99.6 | AI153 | peritrichous flagella |
| M1_GM001475 | 99.6 | VF0532 | HBL |
| M1_GM003520 | 99.6 | CVF558 | Cereolysin O |
| M1_GM000282 | 99.5 | CVF564 | Nonhemolytic enterotoxin NHE |
| M1_GM003678 | 99.4 | CVF567 | Polysaccharide capsule |
| M1_GM001336 | 99.4 | TX186 | InhA1, InhA2, InhA3 |
| M1_GM004556 | 99.4 | TX186 | InhA1, InhA2, InhA3 |
| M1_GM000106 | 99.2 | AI153 | peritrichous flagella |
| M1_GM000921 | 99.2 | AI128 | Bcp pili |
| M1_GM003870 | 99.1 | CVF561 | Hemolysin III homolog |
| M1_GM000631 | 99.1 | CVF560 | Hemolysin III |
| M1_GM000134 | 99 | AI153 | peritrichous flagella |
| M1_GM000284 | 98.9 | CVF564 | Nonhemolytic enterotoxin NHE |
| M1_GM003522 | 98.8 | IA028 | petrobactin |
| M1_GM002987 | 98.7 | IA039 | NEAT-type hemophore-mediated heme uptake system |
| M1_GM001476 | 98.7 | CVF563 | Hemolytic enterotoxin HBL |
| M1_GM003523 | 98.6 | IA028 | petrobactin |
| M1_GM001478 | 98.6 | CVF563 | Hemolytic enterotoxin HBL |
| M1_GM000922 | 98.6 | AI128 | Bcp pili |
| M1_GM000112 | 98.5 | AI153 | peritrichous flagella |
| M1_GM005040 | 98.5 | CVF559 | Cytotoxin K |
| M1_GM002988 | 98.3 | IA039 | NEAT-type hemophore-mediated heme uptake system |
| M1_GM002984 | 98.2 | IA039 | NEAT-type hemophore-mediated heme uptake system |
| M1_GM002967 | 97.8 | IA028 | petrobactin |
| M1_GM000109 | 97.8 | AI153 | peritrichous flagella |
| M1_GM000094 | 97.5 | AI153 | peritrichous flagella |
| M1_GM000128 | 97.2 | AI153 | peritrichous flagella |
| M1_GM000111 | 97.1 | AI153 | peritrichous flagella |
| M1_GM000127 | 97 | AI153 | peritrichous flagella |
| M1_GM000385 | 96.7 | IA028 | petrobactin |
| M1_GM000121 | 96.7 | AI153 | peritrichous flagella |
| M1_GM000136 | 96.5 | AI153 | peritrichous flagella |
| M1_GM003524 | 96.1 | IA028 | petrobactin |
| M1_GM000114 | 96.1 | AI153 | peritrichous flagella |
| M1_GM003679 | 96 | CVF567 | Polysaccharide capsule |
| M1_GM000131 | 95.6 | AI153 | peritrichous flagella |
| M1_GM000093 | 95.6 | AI153 | peritrichous flagella |
| M1_GM003525 | 95.5 | IA028 | petrobactin |
| M1_GM002951 | 95.5 | AI153 | peritrichous flagella |
| M1_GM000135 | 95.4 | AI153 | peritrichous flagella |
| M1_GM000092 | 95.3 | AI153 | peritrichous flagella |
| M1_GM002985 | 95.2 | IA039 | NEAT-type hemophore-mediated heme uptake system |
| M1_GM003695 | 94.8 | CVF567 | Polysaccharide capsule |
| M1_GM000108 | 94.8 | AI153 | peritrichous flagella |
| M1_GM002981 | 94.4 | IA039 | NEAT-type hemophore-mediated heme uptake system |
| M1_GM003694 | 94.1 | CVF567 | Polysaccharide capsule |
| M1_GM000110 | 93.9 | AI153 | peritrichous flagella |
| M1_GM003693 | 93.8 | CVF567 | Polysaccharide capsule |
| M1_GM000096 | 93.8 | AI153 | peritrichous flagella |
| M1_GM000386 | 93.2 | IA028 | petrobactin |
| M1_GM000099 | 93.1 | AI153 | peritrichous flagella |
| M1_GM000384 | 92.7 | IA028 | petrobactin |
| M1_GM000123 | 92.1 | AI153 | peritrichous flagella |
| M1_GM003869 | 92 | CVF567 | Polysaccharide capsule |
| M1_GM000382 | 91.9 | IA028 | petrobactin |
| M1_GM000103 | 91.7 | AI153 | peritrichous flagella |
| M1_GM004562 | 91.3 | TX180 | SM-PLC |
| M1_GM002983 | 91 | IA039 | NEAT-type hemophore-mediated heme uptake system |
| M1_GM000095 | 90.8 | AI153 | peritrichous flagella |
| M1_GM000117 | 90.1 | AI153 | peritrichous flagella |
| M1_GM000104 | 89.5 | AI153 | peritrichous flagella |
| M1_GM000387 | 88.2 | IA028 | petrobactin |
| M1_GM002950 | 88.2 | AI153 | peritrichous flagella |
| M1_GM000118 | 88 | AI153 | peritrichous flagella |
| M1_GM000113 | 86.9 | AI153 | peritrichous flagella |
| M1_GM003680 | 86.8 | CVF567 | Polysaccharide capsule |
| M1_GM002982 | 86.6 | IA039 | NEAT-type hemophore-mediated heme uptake system |
| M1_GM000105 | 86.4 | AI153 | peritrichous flagella |
| M1_GM000383 | 84.3 | IA028 | petrobactin |
| M1_GM003345 | 84 | CVF567 | Polysaccharide capsule |
| M1_GM003696 | 83.8 | CVF567 | Polysaccharide capsule |
| M1_GM003555 | 78.2 | VF0074 | ClpP |
| M1_GM003995 | 78.2 | VF0072 | ClpC |
| M1_GM001458 | 77.9 | CVF572 | Immune inhibitor A metalloprotease |
| M1_GM003684 | 76.1 | CVF186 | Capsule |
| M1_GM002986 | 75.9 | IA039 | NEAT-type hemophore-mediated heme uptake system |
| M1_GM003534 | 75.4 | AI215 | Fibronectin-binding protein |
| M1_GM004021 | 75.4 | CVF827 | EF-Tu |
| M1_GM002794 | 74.4 | CVF228 | Listeria adhesion protein |
| M1_GM004178 | 73.6 | CVF403 | GroEL |
| M1_GM003692 | 71.4 | CVF567 | Polysaccharide capsule |
| M1_GM001140 | 69.4 | VF0074 | ClpP |
| M1_GM005061 | 66.3 | VF0253 | Isocitrate lyase |
| M1_GM003630 | 64.9 | AI128 | Bcp pili |
| M1_GM003610 | 64.2 | VF0144 | Capsule |
| M1_GM004507 | 63.8 | CVF555 | Achromobactin biosynthesis and transport |
| M1_GM002217 | 63.1 | CVF618 | Capsule |
| M1_GM000747 | 62.3 | CVF315 | Mycobactin |
| M1_GM005002 | 62.2 | CVF238 | Lipoate protein ligase A1 |
| M1_GM002547 | 61 | CVF005 | Mg2+ transport |
| M1_GM005501 | 59.8 | CVF305 | Pantothenate synthesis |
| M1_GM003567 | 59.7 | CVF248 | Lipoprotein diacylglyceryl transferase |
| M1_GM002244 | 59.5 | CVF383 | LPS |
| M1_GM005599 | 59.4 | CVF248 | Lipoprotein diacylglyceryl transferase |
| M1_GM002883 | 58.3 | CVF506 | Heme biosynthesis |
| M1_GM002582 | 57.9 | CVF417 | Hemolysin |
| M1_GM000539 | 57.5 | CVF318 | Nitrate reductase |
| M1_GM002739 | 57.5 | AI392 | MOMP |
| M1_GM000744 | 57.4 | CVF553 | Pyochelin |
| M1_GM000484 | 56.4 | VF0403 | Type VII secretion system |
| M1_GM004801 | 56.4 | VF0403 | Type VII secretion system |
| M1_GM003611 | 56.3 | CVF567 | Polysaccharide capsule |
| M1_GM002286 | 55.6 | CVF249 | Lipoprotein-specific signal peptidase II |
| M1_GM001835 | 55.4 | CVF288 | PDIM (phthiocerol dimycocerosate) and PGL (phenolic glycolipid) biosynthesis and transport |
| M1_GM003538 | 54.7 | CVF123 | Streptococcal plasmin receptor/GAPDH |
| M1_GM005117 | 54.6 | CVF861 | T6SS-II |
| M1_GM005207 | 53.8 | VF0356 | Cytolysin |
| M1_GM003682 | 53.6 | CVF775 | Capsule |
| M1_GM002416 | 53.4 | CVF651 | Trehalose-recycling ABC transporter |
| M1_GM005473 | 52.7 | CVF660 | Nucleoside diphosphate kinase |
| M1_GM003683 | 52.5 | CVF854 | Capsule |
| M1_GM001108 | 52.3 | IA009 | thioquinolobactin |
| M1_GM000538 | 52.2 | CVF318 | Nitrate reductase |
| M1_GM002245 | 51.7 | VF0473 | Polar flagella |
| M1_GM003631 | 51.6 | AI128 | Bcp pili |
| M1_GM002700 | 51.3 | VF0169 | SodB |
| M1_GM001364 | 51.1 | CVF411 | Kappa-toxin (collagenase) |
| M1_GM002269 | 51.1 | CVF227 | Fibronectin-binding protein |
| M1_GM004505 | 51 | IA011 | achromobactin |
| M1_GM000600 | 50.9 | CVF253 | LisR/LisK |
| M1_GM005166 | 50.6 | CVF834 | LPS |
| M1_GM005332 | 50.6 | CVF676 | D-alanine-polyphosphoribitol ligase |
| M1_GM002716 | 50.4 | CVF325 | Sigma A |
| M1_GM002127 | 50.4 | CVF516 | ABC transporter |
| M1_GM005246 | 50.2 | CVF651 | Trehalose-recycling ABC transporter |
| M1_GM001914 | 50.1 | VF0388 | kappa-toxin |
| M1_GM004764 | 49.9 | AI128 | Bcp pili |
| M1_GM003884 | 49.6 | CVF667 | RegX3 |
| M1_GM003847 | 49.6 | CVF186 | Capsule |
| M1_GM002895 | 49.5 | CVF149 | Trigger factor |
| M1_GM000881 | 49.5 | VF0388 | kappa-toxin |
| M1_GM002257 | 49.4 | CVF245 | Serine-threonine phosphatase |
| M1_GM004414 | 49.4 | CVF506 | Heme biosynthesis |
| M1_GM000612 | 49.3 | CVF396 | LOS |
| M1_GM004788 | 49.3 | CVF591 | Capsule |
| M1_GM004442 | 49.1 | CVF411 | Kappa-toxin (collagenase) |
| M1_GM003020 | 48.9 | CVF123 | Streptococcal plasmin receptor/GAPDH |
| M1_GM005383 | 48.7 | CVF546 | Phytotoxin phaseolotoxin |
| M1_GM005158 | 48.7 | CVF650 | GPL locus |
| M1_GM005592 | 48.6 | CVF658 | Copper exporter |
| M1_GM004105 | 48.6 | CVF591 | Capsule |
| M1_GM005135 | 48.6 | CVF591 | Capsule |
| M1_GM005545 | 48.6 | CVF833 | Capsule |
| M1_GM005165 | 48.6 | CVF282 | Capsular polysaccharide |
| M1_GM001973 | 48.4 | CVF240 | Oligopeptide-binding protein |
| M1_GM003576 | 48.3 | VF0356 | Cytolysin |
| M1_GM000133 | 48.1 | AI147 | <beta>-GlcNAc |
| M1_GM002861 | 48 | CVF252 | VirR/VirS |
| M1_GM002884 | 48 | CVF506 | Heme biosynthesis |
| M1_GM002122 | 48 | CVF658 | Copper exporter |
| M1_GM005283 | 47.9 | CVF651 | Trehalose-recycling ABC transporter |
| M1_GM000124 | 47.9 | AI153 | peritrichous flagella |
| M1_GM005427 | 47.9 | CVF324 | Mn] superoxide dismutase [Iron-cofactored SOD |
| M1_GM002254 | 47.9 | CVF833 | Capsule |
| M1_GM003800 | 47.9 | IA011 | achromobactin |
| M1_GM000742 | 47.8 | CVF274 | Vibriobactin biosynthesis |
| M1_GM002279 | 47.8 | CVF845 | Pyrimidine biosynthesis |
| M1_GM000745 | 47.6 | CVF849 | Ent siderophore |
| M1_GM000487 | 47.4 | VF0403 | Type VII secretion system |
| M1_GM002902 | 47.4 | CVF494 | LOS |
| M1_GM002216 | 47.4 | VF0361 | Capsule |
| M1_GM002669 | 47.3 | VF0084 | xcp secretion system |
| M1_GM002790 | 47.2 | IA039 | NEAT-type hemophore-mediated heme uptake system |
| M1_GM004582 | 46.9 | VF0356 | Cytolysin |
| M1_GM004226 | 46.8 | CVF839 | Purine |
| M1_GM005370 | 46.8 | VF0171 | LPS |
| M1_GM004571 | 46.7 | VF0356 | Cytolysin |
| M1_GM001586 | 46.5 | CVF333 | MprA/B |
| M1_GM001916 | 46.5 | CVF313 | Magnesium transport |
| M1_GM002143 | 46.4 | AI150 | Pse5Ac7Ac |
| M1_GM002369 | 46.4 | CVF588 | PDH-B |
| M1_GM004142 | 46.4 | CVF591 | Capsule |
| M1_GM005164 | 46.4 | CVF186 | Capsule |
| M1_GM004506 | 46.2 | IA011 | achromobactin |
| M1_GM000751 | 46.1 | AI354 | histone-like protein (Hlp)/laminin-binding protein (LBP) |
| M1_GM004305 | 46 | CVF546 | Phytotoxin phaseolotoxin |
| M1_GM002886 | 46 | CVF506 | Heme biosynthesis |
| M1_GM002565 | 45.9 | CVF588 | PDH-B |
| M1_GM004334 | 45.9 | VF0356 | Cytolysin |
| M1_GM004073 | 45.8 | CVF495 | Exopolysaccharide |
| M1_GM000541 | 45.7 | CVF318 | Nitrate reductase |
| M1_GM003698 | 45.7 | CVF383 | LPS |
| M1_GM001187 | 45.5 | AI144 | ND |
| M1_GM003026 | 45.4 | CVF667 | RegX3 |
| M1_GM001975 | 45.3 | CVF240 | Oligopeptide-binding protein |
| M1_GM004454 | 45.2 | CVF651 | Trehalose-recycling ABC transporter |
| M1_GM005137 | 45 | CVF240 | Oligopeptide-binding protein |
| M1_GM002463 | 45 | SS047 | Dot/Icm |
| M1_GM005468 | 44.9 | AI354 | histone-like protein (Hlp)/laminin-binding protein (LBP) |
| M1_GM005363 | 44.8 | CVF309 | Leucine synthesis |
| M1_GM005093 | 44.8 | CVF760 | Catalase |
| M1_GM001993 | 44.7 | CVF148 | Serine protease |
| M1_GM004439 | 44.7 | CVF573 | Internalin-like |
| M1_GM002830 | 44.7 | CVF335 | (p)ppGpp synthesis and hydrolysis |
| M1_GM000149 | 44.6 | CVF651 | Trehalose-recycling ABC transporter |
| M1_GM000685 | 44.6 | SS179 | HSI-2 |
| M1_GM003279 | 44.4 | CVF252 | VirR/VirS |
| M1_GM001994 | 44.4 | CVF667 | RegX3 |
| M1_GM003857 | 44.3 | CVF762 | Methionine sulphoxide reductase |
| M1_GM005276 | 44.2 | VF0473 | Polar flagella |
| M1_GM005379 | 44.2 | CVF310 | Lysine synthesis |
| M1_GM002280 | 44.1 | CVF845 | Pyrimidine biosynthesis |
| M1_GM001974 | 44.1 | CVF240 | Oligopeptide-binding protein |
| M1_GM002548 | 44.1 | CVF313 | Magnesium transport |
| M1_GM001739 | 44.1 | VF0356 | Cytolysin |
| M1_GM000489 | 43.9 | CVF624 | Type VII secretion system |
| M1_GM000667 | 43.9 | CVF651 | Trehalose-recycling ABC transporter |
| M1_GM002097 | 43.9 | CVF311 | Glutamine synthesis |
| M1_GM005702 | 43.9 | VF0024 | Aureolysin |
| M1_GM005908 | 43.8 | AI354 | histone-like protein (Hlp)/laminin-binding protein (LBP) |
| M1_GM004960 | 43.7 | CVF670 | Post-translocation chaperone |
| M1_GM003322 | 43.7 | CVF313 | Magnesium transport |
| M1_GM005500 | 43.6 | CVF305 | Pantothenate synthesis |
| M1_GM000953 | 43.5 | CVF252 | VirR/VirS |
| M1_GM003866 | 43.2 | VF0169 | SodB |
| M1_GM004429 | 43.1 | CVF803 | T4SS effectors |
| M1_GM005548 | 43.1 | CVF567 | Polysaccharide capsule |
| M1_GM004460 | 43.1 | VF0286 | PhoP |
| M1_GM005368 | 43.1 | CVF520 | LPS O-antigen (P. aeruginosa) |
| M1_GM003458 | 43.1 | VF0356 | Cytolysin |
| M1_GM003681 | 43 | CVF282 | Capsular polysaccharide |
| M1_GM003879 | 42.9 | CVF148 | Serine protease |
| M1_GM000430 | 42.9 | CVF240 | Oligopeptide-binding protein |
| M1_GM003526 | 42.9 | CVF253 | LisR/LisK |
| M1_GM004573 | 42.7 | CVF130 | Sortase A |
| M1_GM002572 | 42.7 | AI142 | lateral flagella |
| M1_GM002120 | 42.7 | AI354 | histone-like protein (Hlp)/laminin-binding protein (LBP) |
| M1_GM005009 | 42.6 | CVF651 | Trehalose-recycling ABC transporter |
| M1_GM001145 | 42.6 | AI396 | M. catarrhalis adherence protein (McaP) |
| M1_GM003956 | 42.6 | CVF834 | LPS |
| M1_GM000125 | 42.4 | SS025 | T3SS |
| M1_GM001942 | 42.3 | VF0473 | Polar flagella |
| M1_GM003198 | 42.2 | VF0414 | RicA |
| M1_GM001932 | 42.1 | CVF396 | LOS |
| M1_GM001116 | 42.1 | CVF521 | Flagella |
| M1_GM005631 | 42 | CVF093 | Thermonuclease |
| M1_GM002323 | 42 | VF0056 | LPS |
| M1_GM003591 | 42 | CVF299 | Accessory secretion factor |
| M1_GM004642 | 42 | CVF737 | ABC transporter for dispersin |
| M1_GM000743 | 41.9 | CVF274 | Vibriobactin biosynthesis |
| M1_GM001109 | 41.9 | IA009 | thioquinolobactin |
| M1_GM000746 | 41.9 | VF0094 | Pyoverdine |
| M1_GM000364 | 41.9 | CVF675 | PdgA |
| M1_GM001127 | 41.7 | CVF588 | PDH-B |
| M1_GM004110 | 41.7 | CVF240 | Oligopeptide-binding protein |
| M1_GM003303 | 41.5 | CVF477 | Enterobactin synthesis |
| M1_GM003612 | 41.5 | CVF186 | Capsule |
| M1_GM004254 | 41.5 | VF0091 | Alginate |
| M1_GM004495 | 41.4 | CVF567 | Polysaccharide capsule |
| M1_GM005167 | 41.3 | CVF282 | Capsular polysaccharide |
| M1_GM002393 | 41.2 | AI117 | type IV pili |
| M1_GM000864 | 41.2 | CVF110 | Capsule |
| M1_GM003109 | 41.1 | CVF667 | RegX3 |
| M1_GM002976 | 41.1 | CVF667 | RegX3 |
| M1_GM005435 | 41 | CVF253 | LisR/LisK |
| M1_GM000962 | 41 | CVF091 | Lipase |
| M1_GM005290 | 41 | CVF573 | Internalin-like |
| M1_GM004093 | 41 | VF0513 | IlpA |
| M1_GM003801 | 40.9 | IA026 | staphyloferrin A |
| M1_GM004387 | 40.9 | CVF520 | LPS O-antigen (P. aeruginosa) |
| M1_GM003102 | 40.9 | CVF775 | Capsule |
| M1_GM000911 | 40.9 | VF0463 | BfmRS |
| M1_GM000630 | 40.8 | CVF528 | Acylhomoserine lactone synthase |
| M1_GM000869 | 40.7 | CVF551 | Pyoverdine |
| M1_GM003853 | 40.7 | CVF834 | LPS |
| M1_GM005901 | 40.7 | VF0356 | Cytolysin |
| M1_GM005260 | 40.6 | CVF667 | RegX3 |
| M1_GM000870 | 40.6 | CVF551 | Pyoverdine |
| M1_GM003255 | 40.5 | CVF667 | RegX3 |
| M1_GM004197 | 40.5 | CVF667 | RegX3 |
| M1_GM000266 | 40.5 | CVF240 | Oligopeptide-binding protein |
| M1_GM000375 | 40.5 | CVF253 | LisR/LisK |
| M1_GM000373 | 40.3 | CVF567 | Polysaccharide capsule |
| M1_GM004529 | 40.3 | AI339 | PEB1 |
| M1_GM005131 | 40.3 | CVF240 | Oligopeptide-binding protein |
| M1_GM000359 | 40.2 | CVF667 | RegX3 |
| M1_GM003677 | 40.2 | CVF333 | MprA/B |
| M1_GM005110 | 40.1 | CVF670 | Post-translocation chaperone |
| M1_GM000706 | 40.1 | CVF670 | Post-translocation chaperone |
| M1_GM002791 | 40.1 | IA028 | petrobactin |
| M1_GM000991 | 40.1 | CVF396 | LOS |
| M1_GM004984 | 40.1 | CVF506 | Heme biosynthesis |
| M1_GM003685 | 40.1 | CVF186 | Capsule |
| M1_GM005615 | 40 | CVF667 | RegX3 |
| M1_GM004540 | 40 | CVF667 | RegX3 |
| M1_GM004372 | 40 | AI143 | ND |
| M1_GM005882 | 40 | CVF649 | MymA operon |
| M1_GM005878 | 40 | CVF649 | MymA operon |
| M1_GM005891 | 40 | CVF649 | MymA operon |
| M1_GM004741 | 40 | CVF760 | Catalase |
| M1_GM004104 | 40 | CVF591 | Capsule |
